# Unraveling the Nature of Antibiotics: Is It a Cure or a New Hurdle to the Patient Treatment?

**DOI:** 10.7759/cureus.23955

**Published:** 2022-04-08

**Authors:** Sai Sreeya Gude, Shravya Venu Gopal, Harshita Marasandra Ramesh, Sravya Vuppalapati, Nikhil Chowdary Peddi, Sai Sravya Gude

**Affiliations:** 1 Internal Medicine, Guntur Medical College, Guntur, IND; 2 Internal Medicine, Kasturba Medical College, Mangalore, Mangalore, IND; 3 Internal Medicine/Pediatrics, Kasturba Medical College, Mangalore, Mangalore, IND; 4 Pediatrics, PES Institute of Medical Sciences and Research, Kuppam, IND; 5 Pediatrics, Guntur Medical College, Guntur, IND

**Keywords:** rapid microbial diagnostics, antibiotic stewardship programs, antimicrobial susceptibility testing, rational use of antibiotics, antimicrobial resistance

## Abstract

Antimicrobial resistance is an increasing problem worldwide that has been exacerbated by antibiotic misuse worldwide. Growing antibiotic resistance can be attributed to as well as leads to severe infections, complications, prolonged hospital admissions, and higher mortality. One of the most important goals of administering antimicrobials is to avoid establishing antibiotic resistance during therapy. This can be done by drastically lowering worldwide antimicrobial usage, both in present and future. While current management methods to legislate antimicrobials and educate the healthcare community on the challenges are beneficial, they do not solve the problem of attaining an overall reduction in antimicrobial usage in humans. Application of rapid microbiological diagnostics for identification and antimicrobial susceptibility testing, use of inflammation markers to guide initiation and duration of therapies, reduction of standard antibiotic course durations, individualization of antibiotic treatments, and dosing considering pharmacokinetics are all possible strategies to optimize antibiotic use in everyday clinical practice and reduce the risk of inducing bacterial resistance. Furthermore, to remove any impediments to proper prescribing, strategies to improve antibiotic prescribing and antibiotic stewardship programs should enable clinical reasoning and enhance the prescribing environment. In addition, the well-established association between antimicrobial usage and resistance should motivate efforts to develop antimicrobial treatment regimens that facilitate the evolution of resistance. This review discusses the role of antibiotics, their current application in human medicine, and how the resistance has evolved to the existing antibiotics based on the existing literature.

## Introduction and background

Antibiotics have changed medicine in numerous ways, saving innumerable lives; their discovery was a watershed moment in human history [1]. Unfortunately, the widespread use of these miracle medications has resulted in the rapid emergence of resistant strains [1]. The possible development of tolerance or resistance to any treatment drug from the moment it is first used jeopardizes its effectiveness [1]. The most severe effect of antibiotic use, by any measure, is the emergence of resistant strains; this has motivated ongoing efforts to establish control over antibiotic use [1].

Antibiotic resistance can be caused by overuse of antibiotics, inappropriate prescribing, poor compliance, extensive agricultural use, poor infection control in hospitals and clinics, and the availability of few new antibiotics [2]. Antibiotic resistance can be either inherent or acquired. Inherent antibiotic resistance refers to an organism's natural resistance to antibiotics. For example, enterococci are inherently resistant to cephalosporins, while nafcillin is naturally resistant to *Pseudomonas aeruginosa* [3]. Bacteria adapt to antibiotic "attacks" through two key genetic strategies: 1) mutations in the gene(s) generally connected with the compound's mechanism of action and 2) acquisition of foreign DNA coding for resistance determinants by horizontal gene transfer [4].

Antibiotic-resistant bacterial infections are projected to cause over 33,000 fatalities in Europe each year, and antimicrobial resistance is expected to generate 10 million deaths worldwide by 2050 [5,6]. The complexity of the processes that accord with the emergence of resistance cannot be overstated in the case of antimicrobial agents, and an absence of basic knowledge on these topics is one of the hurdles for the failure of significant progress in the effective prevention and control of resistance development [1]. Antibiotic resistance can be reduced or avoided by increasing knowledge and awareness, strengthening ability through observation and research, reducing infection rates, and optimizing the use of antimicrobial medications [2].

## Review

Role of antibiotic resistance in medicine 

Antibiotic stewardship programs, a collection of strategies, policies, guidelines, or tools aimed at improving antibiotic use, aim to reduce overall antibiotic consumption and inappropriate antibiotic use, which are essential measures to reduce the emergence of resistant bacteria [7,8]. Recent studies have revealed that global antibiotic usage in human medicine is rising. However, incorrect antibiotic prescribing is still expected; it is estimated that up to half of all antimicrobials administered to people are unnecessary [5,9]. 

The processes, emergence, and diffusion of antibiotic resistance in hospital and community settings have been mapped out using lessons learned from 80 years of clinical antibiotic usage and development [10]. The genomic era's scientific breakthroughs have been crucial in establishing links between antibiotic resistance in environmental microorganisms and human diseases. Microbial natural products account for most therapeutic antibiotics [11]. As a result, natural conditions where antibiotic biosynthesis is shared are the source of the most clinical antibiotic resistance [1]. Indeed, the aminoglycoside-modifying and tetracycline-inactivating enzymes are believed to have evolved from the production of aminoglycoside and tetracycline antibiotics, respectively [12-14].

Because well-documented biochemical or genetic alterations fail to explain processes behind antibiotic resistance adequately, it is becoming clear that we need to look at newer, nontraditional mechanisms like epigenetic control. Epigenetic alteration's biological importance in influencing gene expression and other cellular processes is becoming more well acknowledged. Both prokaryotes and eukaryotes are discovering new epigenetic changes, such as phosphorothioation in the bacterial DNA backbone and acetylation of cytidine in eukaryal mRNA [15-17]. Alterations in eukaryotic mRNAs have been shown to control cellular activities, so it is not too far-fetched that similar modifications in bacterial transcripts may be connected to essential roles in the bacterial life cycle [17,18].

The study conducted by Peddi and Latha in a rural tertiary hospital setting published in 2021 suggested that *Staphylococcus aureus* (16.7%) was the most common Gram-positive isolate [19]. It was most sensitive to amikacin (50.0%) and cotrimoxazole (50.0%) and least sensitive to penicillin (0%), tetracycline (0%), tobramycin (0%), and ceftriaxone (0%) [19]. The most successful antibiotics in this clinical context, according to that study, were imipenem (51.4%), meropenem (51.4%), and piperacillin + tazobactam (51.4%), and most of the organisms were resistant to tobramycin, tetracycline, and penicillin [19]. The study also found two methicillin-resistant *Staphylococcus aureus* (MRSA) organisms, both were susceptible to erythromycin, and one organism was sensitive to cotrimoxazole, gentamicin, amikacin, and ceftazidime [19]. This shows the trend in antibiotic resistance, which is overgrowing [19].

Because hospitals vary in size, geography (rural, suburban, urban), teaching (vs community), staff antibiotic prescription tendencies, presence or absence of full-time infectious disease physicians, resistance trends, and other factors, antibiotic stewardship programs (ASPs) must differ. One solution is not the answer to all problems. In one hospital, what is a successful ASP intervention is unsuccessful in another [20]. Customizing ASP treatments to the hospital's particular collection of antibiotic use-related problems is the responsibility of the ASP ID team leader and clinical infectious disease (ID)-trained PharmD employees. In antimicrobial stewardship programs (ASPs), the findings from prospective audits and the effectiveness of various ASP interventions are examined and changed. Future audits can help uncover unsuccessful initiatives and recommend changes or altogether new ways [20].

Rapid microbial diagnostics

Infections in the bloodstream are the primary cause of illness and death [21]. Therefore, it is of paramount significance to speed up the identification and susceptibility testings and adjust the appropriate antibiotic medication to improve patient outcomes [21,22]. The standard method to detect microorganisms comprises overnight agar medium subcultures from positive blood culture bottles, which might take up to 24-48 hours for results. However, direct injection from positive blood cultures into automated systems can decrease the identification time [21,23].

Novel techniques such as matrix-assisted laser desorption/ionization time-of-flight mass spectrometry (MALDI-TOF MS) have revolutionized clinical microbiology [22]. The combination of MALDI-TOF MS-based antimicrobial susceptibility testing (AST) and MALDI-TOF MS identification plays a significant role in the choice of antibiotic treatment [22]. Rapid identification of microbes from positive blood cultures can be achieved by two methods using the MALDI-TOF MS: direct identification and identification after a short period of subculture in a solid medium [24]. In direct identification methods, the blood culture suspension is treated with a detersive agent and centrifuged, and protein extraction is carried out using formic acid and ethanol. It is then subjected to MALDI-TOF MS analysis. This approach allows very quick identification (20-40 minutes) [24].

Direct-on-target microdroplet growth assay (DOT-MGA) is a unique method of MALDI-TOF MS to detect antibiotic sensitivity [25]. The basis of this methodology is as follows: to execute DOT-MGA, microbes incubated with or without antibiotics in nutrient broth as microdroplets on the target areas. At varying antibiotic concentrations, the microbial growth in each of the target plates was examined to see if MALDI-TOF MS could successfully identify each microbial spot. Microbes could be classified as sensitive or resistant, and minimal inhibitory concentration (MIC) could be computed based on the antibiotic concentration in each microdroplet [26].

Initially, Idelevich et al. developed this technique to assess carbapenem susceptibility in* Klebsiella pneumoniae *and *Pseudomonas aeruginosa* by incubating microdroplets of bacterial suspension with or without antibiotics on a MALDI target surface for a short time to detect bacterial growth and determine its antibiotic sensitivity [27]. This technique promises rapid and reliable AST. It also provides information about the mechanism of resistance [27].

Current applications

Clustering techniques demonstrate the value of using the complete spectrum and effective classifiers to determine species/strain types that are pretty similar [28]. For example, de Bruyne et al. showed that identical *Leuconostoc*, *Fructobacillus*, and *Lactobacillus* species could be recognized to species level [29].

Clonality

Spectral features can also be used to distinguish microbes based on their clonality, such as methicillin-resistant *Staphylococcus aureus *(MRSA), vancomycin-resistant *Enterococcus* (VRE), and β-lactamase strains [28]. Also, another automated system, namely the Vitek-2 system, provided reliable AST results in positive blood cultures of Gram-negative rods and Gram-positive cocci [21,23]. Multiplex polymerase chain reaction (PCR), despite delivering accurate results, has its drawbacks such as being expensive, simultaneous processing of less number of samples, and a limited range of pathogens [24]. The limitations of MALDI-TOF MS include AST testing of polymicrobial cultures and detection of slow-growing organisms [24]. It is also quite challenging to detect fungi using the same [24,29].

Inflammation Biomarkers

Antibiotic use and the emergence of resistance have a well-established causal link. The prevalence of multiresistant organisms is higher in individuals receiving long-term antibiotic therapy, and increased fungal infections even in immunocompetent individuals have become a major source of worry [30]. Antibiotic therapy of viral diseases or noninfectious inflammatory disorders (NIID) that is unwarranted results in higher expenses, negative medication responses, and antibiotic resistance. Many microbiological procedures, such as cultures, serology, and PCR, have their own set of constraints such as difficulty obtaining a suitable biological specimen, low sensitivity, increased expenses, and long turnaround times, which hinder their impact on early decision-making [31].

Biomarkers that represent the immune response of the host may be an appealing technique for predicting the genesis of an inflammatory condition and are intended to offer an assessment of the severity of infection or forecast a difficult course to aid in the selection of the best therapeutic approach and the most appropriate care setting as well as assist the doctor in deciding whether to start or continue antibiotic treatment [31,32]. Whether mild or severe, all bacterial infections cause the acute phase of inflammation to produce cytokines and proteins. However, in some viral infections, cytokine and C-reactive protein (CRP) levels might be quite high [33].

White blood cells (WBCs) or absolute neutrophil count (ANC), leukocyte surface markers such as CD64 and CD35, soluble diagnostic biomarkers like C-reactive protein (CRP) and procalcitonin, and cytokines such as interleukin (IL)-6 and tumor necrosis factor (TNF)-α can be utilized as a diagnostic tool for follow-up or to choose patients who are likely to benefit from a specific treatment. Biomarkers can also be used during therapy follow-up as early indicators of efficacy or treatment toxicity [31,32,34].

The two most commonly used biomarkers are CRP and procalcitonin [32]. CRP is a pentraxin-like acute phase protein produced primarily in the liver, and its production depends on interleukin-6 [31]. In children, CRP levels are commonly used to alter the course of medication and optimize antibiotic therapy [32]. But, for bacterial infections, CRP or WBCs lack specificity which is explained by the heterogeneity of infectious agents and the complex interaction between various pro- and anti-inflammatory mediators of the host response countering invading pathogens during systemic infections, which varies based on the duration, type, extent, and location of the underlying infection [35].

Procalcitonin-Guided Antibiotic Therapy

Procalcitonin (PCT) is a rediscovered biomarker that meets several criteria, particularly when compared to other widely used biomarkers showing greater diagnostic accuracy for various illnesses. PCT is proven helpful in detecting sepsis early and monitoring the antimicrobial treatment plan. PCT can be a beneficial tool for antimicrobial stewardship, and its use can effectively result in a significant decline in antimicrobial therapy administration [36]. Numerous randomized controlled studies have looked into the use of PCT to help with the induction of antibiotic therapy and duration decisions [35].

PCT is synthesized in response to endotoxins or mediators, interleukin (IL)-1β, tumor necrosis factor (TNF)-α, and IL-6, during bacterial infections and has a strong correlation with the severity and extent of bacterial infections [35]. In addition, serum PCT levels are higher in bacterial, fungal, and parasitic infections than in viral illnesses or noninfected patients, making PCT an antibiotic treatment guide [37].

Compared to C-reactive protein, the biomarker, procalcitonin, shows a more vital and faster modulation for the severity of bacterial infection. As a result, a reasonable decline in procalcitonin concentrations may aid in the earlier discontinuation of antibiotic usage. Furthermore, according to all evidence, procalcitonin-guided treatment can shorten antibiotic treatment times. This is possible even in a relatively brief antibiotic treatment period. However, it is unclear whether the procalcitonin assay will be cost-effective [38].

The average concentration of PCT in human serum is less than 0.1 ng/ml. Individuals with clinical symptoms of lower respiratory tract infection (LRTI) and patients with clinical signs of sepsis have different PCT strategies. Patients with LRTI can stop taking antibiotics if their PCT level is less than 0.25 ng/ml, while patients with sepsis can stop taking antibiotics if their PCT level is less than 0.50 ng/ml. Furthermore, a drop in PCT of 80% or more from the peak level necessitates antibiotic withdrawal for both situations. Every 24 to 48 hours, PCT levels are usually taken. If the baseline PCT level is below the antibiotic termination threshold, a repeat measurement within six to 24 hours is indicated to account for patients who have a late PCT peak. It is vital to remember that PCT levels should always be evaluated using clinical evaluations and other laboratory indicators. Antimicrobial therapy decisions should not be based solely on PCT levels [39]. The contribution of a local guideline advising against the use of antibiotics for confirmed cases of COVID-19 with PCT <0.25 ng/ml was shown in an observational analysis, which resulted in reduced antibiotic usage with no negative influence on the 28-day outcome [40].

Meta-analysis findings indicated that patients randomized to PCT procedures had much lower antibiotic exposure and related side effects, with a reduction in antibiotic initiation from 86% to 72% and overall exposure from 8.1 to 5.7 days. Antibiotic side effects reduced from 22.1% to 16.3% [41]. While a majority of the studies have focused on the impact of PCT guidance on antibiotic usage, a recent major trial from the Netherlands found that PCT-guided antibiotic therapy was associated with decreased mortality; however, it did not affect the length of time spent in the ICU or the hospital [42].

The BRAHMS LUMI (BRAHMS Aktiengesellschaft, Hennigsdorf, Germany) test, employed in older diagnostic investigations to detect PCT levels, has a low sensitivity and is less likely to be utilized in clinical settings. Therefore, newer PCT testing solutions have recently been introduced, including the KRYPTOR (BRAHMS Aktiengesellschaft, Germany); VIDAS system (bioMérieux, France), Liaison BRAHMS PCT (DiaSorin, Italy), and Elecsys' BRAHMS PCT (Roche Diagnostics, Basel, Switzerland) [35] have exhibited a high level of similarity and correlation with the well-established BRAHMS KRYPTOR technique [43].

Limitations

Every PCT measurement has limitations, including false-positive and false-negative outcomes. Different infections may elicit different responses, resulting in varying increases in circulating PCT levels. For example, patients with pneumococcal community-acquired pneumonia (CAP) had high PCT levels; however, this was not the case in CAP caused by atypical organisms like mycoplasma. In addition, antimicrobial pretreatment may influence PCT levels, resulting in reduced levels [35]. Patients with comorbid diseases, such as renal dysfunction, malignancy, or congestive heart failure (CHF), should have their PCT levels carefully assessed, as these disorders can raise PCT levels even when there is no bacterial infection [39].

Newer Biomarkers

Newer biomarkers including the soluble triggering receptor expressed on myeloid cells-1 (sTREM-1), soluble urokinase-type plasminogen receptor (suPAR), proadrenomedullin (ProADM), and presepsin indicate that they could play a role in future clinical advances, whether as diagnostic tests, categorization of patients by kind of injury or severity, or assessment of therapeutic activity and efficacy, and during patient follow-up. Presepsin is the most sensitive and specific of the four biomarkers, and it may help distinguish systemic inflammatory response syndrome (SIRS) from sepsis caused by bacterial infection [32].

Micro-RNAs (miR) are a new class of biomarkers recently found. MiRs are tiny molecules (approximately 20 nucleotides) found in eukaryotic cells that modulate post-transcriptional regulation as biologic regulators. Reverse transcription-polymerase chain reaction (RT-PCR) and quantitative PCR can be used to determine their expression. MiR-150, miR-182, and miR-342-5p are the three most dysregulated miRs; miR-150 inhibits lymphocyte immune response development and can be used as an early diagnostic or prognostic marker [32].

Duration of Antibiotic Therapies to Reduce Bacterial Resistance

Traditional antibiotic therapy durations are based on the fact that a week equals seven days, which is why tried-and-true antibiotic regimens are seven to 14 days long. As a result, more time was not better. Furthermore, patients who took longer courses experienced much more significant adverse effects, indicating that longer is worse. Each additional day of antibiotic medication elevated the probability of an unfavorable event by a startling 5%. Importantly, and in line with prior randomized control trials (RCTs), patients who received lengthier antibiotic courses did not have higher survival rates, lower readmission rates, or fewer emergency department visits [44]. Current concerns are primarily about developing resistance in common commensal bacteria rather than in the bacteria that cause infections. There is growing evidence that the longer an antibiotic is used, the greater the development of antibiotic resistance, increasing the risk of resistance in subsequent infections [45,46].

A recent study found that in patients who had previously used antibiotics intermittently, the therapy was less effective than in those who had only used them occasionally [47,48]. This shows that frequent use reduces antibiotic effectiveness and may lead to individualized resistance, increasing the risk of treatment failure in the future. Antimicrobial use should be minimized to extend the efficacy of current antimicrobials within the human population [47,48]. To achieve the desired therapeutic impact, only antimicrobials that the infecting pathogen is susceptible to should be used, and they should be used for the shortest duration and in the smallest dose possible [49].

This can be accomplished by prescribing the shortest course of treatment (or the total number of pills), even if it requires the pharmacist to break the box. For example, when antibiotics are administered, the duration (or the number of medications) should be indicated on the prescription, allowing the pharmacist to deliver only the amount of tablets or capsules needed (even if this involves breaking the antibiotic pack), preventing overuse of antibiotics [45].

Choosing the Right Antibiotic Class

An excessively homogeneous usage (a few compounds/antibiotic classes) may enhance selection pressure and encourage antimicrobial resistance spread [5,50]. In addition, single-drug therapy may favor the formation of resistance to infections caused by some microorganisms (Bauernfeind et al., 1995) [51]. Similarly, vancomycin-resistant enterococci (Hsueh et al., 2005b), extended-spectrum β-lactamase (ESBL)-producing Enterobacteriaceae (Meyer et al., 2010), carbapenemase-producing Gram-negative bacteria (Furtado et al., 2010), multiresistant *Pseudomonas aeruginosa* (Polk et al., 2004; Hsueh et al., 2005a; Weng et al., 2011), and *Acinetobacter baumannii* (Hsueh et al., 2005a; Meyer et al., 2009) have also been linked to increased use of antimicrobials in a single institution [52-58].

Over time, MRSA bacteria have become resistant to all β-lactam antibiotics, macrolides, and aminoglycosides. Enterococci have evolved resistance to vancomycin and ampicillin over time. The usage of cephalosporins has been linked to hospital-acquired infections caused by enterococci [59]. In addition, enterocolitis has been connected to prophylactic cephalosporins in operations, and their use for this purpose is currently prohibited in specific at-risk patient categories [60]. The WHO's Access, Watch, and Reserve (AWaRe) antibiotic categorization was introduced to combat the emergence of antimicrobial resistance (AMR). It divides antibiotics into three stewardship groups: access, watch, and reserve, emphasizing the importance of antibiotics' optimal uses and potential for antimicrobial resistance. The access category comprises empiric therapy options for specific infectious disorders as a first- or second-choice alternative. Antibiotics in the watch group have a more significant resistance potential. Most of the top priority drugs are used as the first- or second-line empiric therapy choices for certain infectious disorders. Antibiotics in the reserve group should be used only for proven or suspected illnesses caused by multidrug-resistant organisms [61].

Antibiotics should be administered only when necessary, and the medicine chosen should be the most narrow-spectrum agent that will be successful. Appropriate usage entails selecting the right antibiotic and the correct dose and duration, all of which might impact the development and carriage of resistance microbes [59]. Antibiotic cycling, combination therapy, and avoiding wide spectrum and last-resort antibiotics wherever possible have all been applied to prevent the evolutionary pressure that drives resistance [60]. Recommendations on the first- and second-choice antibiotics to be used for the treatment of the most common and severe clinical infections according to the AWaRe principles are provided on the AWaRe portal (https://aware.essentialmeds.org/).

Choosing the Right Dosage

Antibiotic resistance is a public health concern, and the fast rise of resistant bacteria has raised public awareness of the issue. Despite this, antibiotic solid usage laws are missing, and the research of new antibiotics is becoming increasingly costly and complicated. Furthermore, antibiotic resistance can be chosen during antibiotic therapy, as it is widely known that selection takes place both at the site of infection and in the commensal flora [62-64]. A neglected study topic has been the relationship between medication dose and resistance development. Therefore, dosage regimens should be chosen carefully to ensure that current and novel antibiotics have a long shelf life. In addition, pharmacokinetics/pharmacodynamic (PK/PD) features that inhibit the appearance of preexisting or newly created mutants should be considered while choosing dosing regimens [65].

It is logical to expect, and it has previously been demonstrated in vitro, that greater antibiotic dosages are linked with a lower incidence of resistance development than low doses. This linkage is because increased dosages result in higher drug concentrations at infection sites, such as abscesses, empyemas, and other diseases with many bacteria. As a result, higher dosages are likely to result in less de novo resistance [50,58,66-68]. It has been demonstrated that when children in the community are treated with β-lactam antibiotic dosages that are lower than the recommended standard doses (OR, 5.9; CI, 2.1-16.7; P=0.002), the carriage rate of penicillin-resistant *Streptococcus pneumoniae* (PRSP) increases [69].

Antibiotic resistance develops in the patient due to delays in starting medication, low dosages, and extended intervals between doses (inadequate pharmacokinetics). Notably, very low antibiotic doses can be selected for low-level resistant mutants, acting as stepping stones to high-level resistance [70,71]. Understanding how low-dose and/or long-term antibiotic use promotes bacterial resistance can positively improve physicians' antibiotic prescribing practices [72]. Thus, to avoid resistance, the "maximum tolerated dosage" achievable for the shortest length of therapy would be preferred to the "minimally effective dose" for more extended periods of treatment, which is now the dominant idea [66].

## Conclusions

The usage of antibiotics and the rise of resistance are unquestionably connected. However, with proper antibiotic regimens, the establishment of resistance can be prevented or at least slowed to some extent. Because bacteria's usual reaction to antibiotic exposure is to generate genetic diversity to withstand the antibiotic's effects and because man's normal flora contains species resistant to every antibiotic, antibiotic usage will always result in the formation of antibiotic resistance. All one can hope for is reducing the harm caused by antibiotic use. We know that we can minimize antibiotic prescription in many illnesses that are now being treated needlessly without jeopardizing the health of our patients. Antimicrobial regimens should be tailored to provide therapeutic effectiveness while simultaneously minimizing the formation and spread of resistance. Careful clinical reasoning is the foundation for making the best antibiotic therapy decisions. We can optimize antibiotic treatment by prescribing the right antibiotic, right dose, application, and correct therapy duration.
